# Identification of FactorsInfluencing the Puumala Virus Seroprevalence within Its Reservoir in aMontane Forest Environment

**DOI:** 10.3390/v6103944

**Published:** 2014-10-23

**Authors:** Bryan R. Thoma, Jörg Müller, Claus Bässler, Enrico Georgi, Anja Osterberg, Susanne Schex, Christian Bottomley, Sandra S. Essbauer

**Affiliations:** 1 Bundeswehr Institute of Microbiology, Neuherbergstr. 11, 80937 Munich, Germany; E-Mails: bryanthoma@bundeswehr.org (B.R.T.); enrico1georgi@bundeswehr.org (E.G.); anja.osterberg@web.de (A.O.); susanneschex@yahoo.com.au (S.S.); 2 Bavarian Forest National Park, Freyunger Str. 2, 94481 Grafenau, Germany; E-Mails: joerg.mueller@npv-bw.bayern.de (J.M.); claus.baessler@npv-bw.bayern.de (C.B.); 3 Chair for Terrestrial Ecology, Department of Ecology and Ecosystem Management, Technische Universität München, Hans-Carl-von-Carlowitz-Platz 2, 85354 Freising, Germany; 4 University of Rostock, Universitätsplatz 1, 18055 Rostock, Germany; 5 MRC Tropical Epidemiology Group, London School of Hygiene and Tropical Medicine, Keppel St, London WC1E 7HT, UK; E-Mail: christian.bottomley@lshtm.ac.uk

**Keywords:** bank vole, Puumala virus, climate, population, forest, risk prediction, GLMEM

## Abstract

Puumala virus (PUUV) is a major cause of mild to moderate haemorrhagic fever with renal syndrome and is transmitted by the bank vole (*Myodes glareolus*). There has been a high cumulative incidence of recorded human cases in South-eastern Germany since 2004 when the region was first recognized as being endemic for PUUV. As the area is well known for outdoor recreation and the Bavarian Forest National Park (BFNP) is located in the region, the increasing numbers of recorded cases are of concern. To understand the population and environmental effects on the seroprevalence of PUUV in bank voles we trapped small mammals at 23 sites along an elevation gradient from 317 to 1420m above sea level. Generalized linear mixed effects models(GLMEM) were used to explore associations between the seroprevalence of PUUV in bank voles and climate and biotic factors. We found that the seroprevalence of PUUV was low (6%–7%) in 2008 and 2009, and reached 29% in 2010. PUUV seroprevalence was positively associated with the local species diversity and deadwood layer, and negatively associated with mean annual temperature, mean annual solar radiation, and herb layer. Based on these findings, an illustrative risk map for PUUV seroprevalence prediction in bank voles was created for an area of the national park. The map will help when planning infrastructure in the national park (e.g., huts, shelters, and trails).

## 1. Introduction

**Figure 1:**
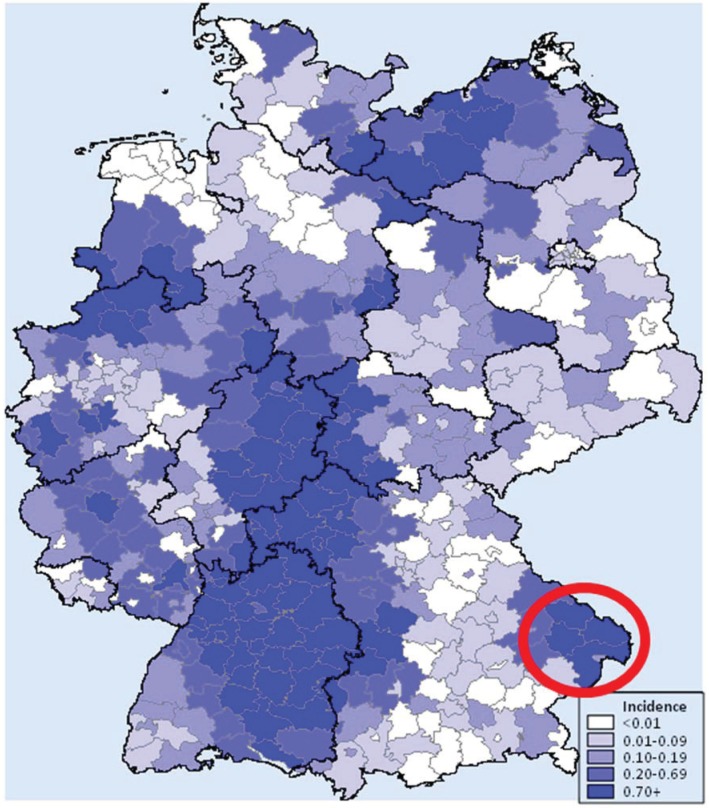
Cumulative incidence (2001–2013) of clinically apparent hantavirus infections per 100,000 inhabitants population in administrative districts of Germany [11]. The study region of Lower Bavaria is marked in red.

Puumala virus (PUUV, genus *Hantavirus*, family *Bunyaviridae*) is the most common hantavirus in Europe. PUUV causes haemorrhagic fever with renal syndrome (HFRS) and nephropathia epidemica (NE), a mild to moderate form of HFRS. It is endemic in several European countries with varying numbers of recorded cases in humans each year [1,2]. The natural host for PUUV is the bank vole (*Myodes* (*M.*) *glareolu*s). Infections in bank voles are persistent and seem to be asymptomatic but may reduce winter survival [3]. Transmission of the viruses to humans occurs mostly by inhalation of virus‑contaminated aerosols, or through direct contact with rodents’ skin lesions and by bites. Since 2001, clinically apparent hantavirus infections are notifiable diseases in Germany according to the German Federal Infection Protection Act. Between 2001and the autumn of 2013, a total of 8674clinically apparent infections have been recorded. Annual case numbers in Germany were very variable from 72 in 2006 up to 2824 in 2012. Years with a high number of cases were 2005 (n = 447), 2007 (n = 1687), 2010 (n=2016), and 2012 (n=2824) [4,5,6,7,8,9,10,11]. The higher than usual case numbers were also recorded in neighbouring countries in2005. However, in general, annual patterns of registered human hantavirus infections in Europe may differ considerably [4,5,12]. Seasonal peaks of human infections usually occur in the summer months, but peaks can also occur in winters that precede outbreak years [7,12]. Most hantaviral infections in Germany are recorded in the federal states of Baden‑Wuerttemberg (53.5%), Bavaria (17.1%), and North Rhine-Westphalia (10.8%). Several regions within these and other federal states are known to be endemic for hantaviruses, e.g., the Swabian Alp in Baden-Wuerttemberg, the Spessart region, Lower Bavaria in Bavaria, the area around Muenster, the Teutoburg Forest, West-Thuringia, and North Hesse (for details see Figure 1) [8,9,13,14,15,16].

Since 2004, the area of Lower Bavaria, Southeast Germany, has been a focus of PUUV-monitoring [9,14,15,17,18]. Recorded cases of human PUUV hantavirus infections in Lower Bavaria were reported for the first time in 2002 (n=3); since then the number of cases was highest in 2004 (n= 38) and 2007 (n=47) cases. The increasing trend in the human hantavirus incidence has been observed in other parts of Germany and in other European countries since2007. In 2010,a 10-fold increase relative to the previous year was reported in several parts of Germany [1,9]. Remarkably, in 2010 seventy-eight PUUV infections were registered in Lower Bavaria, and 47 clinically apparent cases were recorded in the surrounding Bavarian Forest National Park (BFNP) [11]. In 2012, an even higher incidence of hantavirus infections was recorded with 108 cases in close vicinity to the BFNP (for details see Figure 1) [11].

In the past decade some studies have investigated hantavirus dynamics and its emergence in Europe. The identification of risk factors is crucial for predicting the risk of PUUV-transmission to humans and PUUV outbreaks, and also understanding the observed annual, seasonal oscillations and spatial variation. In particular, environmental and climatic conditions [12] are likely to be important predictors of PUUV in the bank vole populations.

It has been suggested that bank vole density and PUUV seroprevalence in bank vole populations greatly influence the risk of human PUUV disease [19,20,21]. Furthermore, changes in rodent populations have an impact on PUUV infections in the bank vole. In particular, older and sexually active male voles with higher body mass index have a higher virus prevalence [19,22,23,24,25,26].The trapping index can be used as a parameter for studying and comparing rodent population densities and therefore the impact of rodent populations on the prevalence of PUUV [27,28]. 

The influence of biodiversity on the emergence of diseases is controversial and has been extensively discussed [29,30,31]. However, to our knowledge the impact of biodiversity on the emergence of European hantaviruses has not been investigated so far.

Virus stability might be influenced by a number of environmental factors, e.g., UV radiation, humidity, or temperature. For example, Hantaan virus is inactivated rapidly by UV radiation [32]; therefore, PUUV infection might be less common in areas with a high environmental solar radiation. Further, *in vitro* studies have revealed that a low temperature directly increases the stability of the virus particles [33]. Thus, the virus might be more easily transmitted in colder environments [12,34,35]. 

The micro-/habitat structure is crucial for the fitness and survival of bank voles [36,37,38] and might influence the prevalence of PUUV in the host. Densely covered herb- and shrub layers serve as a preferred habitat for *M. glareolus* by providing shelter and food. The bank vole population size may be positively related to the coverage and density of these layers [39,40,41]. The positive association between individual components of the herb- and shrub layer (e.g., blueberry, lingon berry, and tree lichens) with bank vole density, and PUUV occurrence in voles has been demonstrated using principal components analysis [42]. Further, deadwood may serve as a favourable microhabitat for bank voles and PUUV foci may be associated with high amounts of deadwood [43,44]. Some PUUV outbreaks have been linked with a high fructification of European beech and pedunculate oak in the preceding year. An abundance of food for bank voles (“mast”) may lead to population increases, and, as a consequence, higher PUUV prevalence and transmission [21,34,35,45,46,47,48].

Predictions of PUUV prevalence based on environmental, climatic, and human demographic parameters are scarce. One study used data from Finland and Belgium, and the mean relative prediction error of the model was 34% and 40%, respectively [48]. However, the model might not provide accurate predictions of PUUV prevalence in humans in different bio-/geographic and climatic regions [49].

In general, bank voles have small territories between 450 to 2000 square meters (m^2^) [39,50,51,52,53,54,55,56]. So far, most studies have modelled the risk of PUUV transmission or outbreaks at the country or state level [34,40,48,49,57,58]. However, considering the small habitats of bank voles, and, in Germany, some very circumscribed PUUV hotspot areas within different biogeographic regions(e.g., the North German Plain, uplands, river valleys, linear hills, high mountain ranges such as the Bavarian Forest [59]) we chose to study PUUV at a smaller scale. The current study uses data collected on rodents and associated pathogens (e.g., PUUV and Rickettsiae) along an altitude gradient in the BFNP together with environmental and climatic data generated in the BIOKLIM (Biodiversity and Climate Change Project) project [60,61]. 

The overall aim of this study was to examine the potential link between environmental and climatic factors and the seroprevalence of PUUV in the bank vole population in our study region. We collected data on the following factors: (I) trapping index as a measure of host density, (II) small mammal species diversity, (III) mean annual temperature, (IV) mean annual solar radiation, (V) forest herb layer coverage, (VI) forest shrub layer coverage, (VII) forest deadwood layer coverage, and (VIII) beech tree coverage. We generated an illustrative risk map of predicted PUUV seroprevalence in bank voles foran area within the BFNP.

## 2. Results and Discussion

### 2.1. Animal Species, Diversity, Abundance and PUUV Seroprevalence

Small mammals were captured at 23 trapping sites along an altitude gradient ranging from 317 to 1420 m above sea level (asl) and mostly dispersed in the BFNP between 2008 and 2010. The trapped animals included 674 individuals of five rodent species, some insectivores and one carnivore. In summary, 353 *M. glareolus* , 262 *Apodemus*(*A*.) spp. (*A. flavicollis* and *A. sylvaticus*), 35 *Microtus agrestis*, eight *Glis glis*, one *Mustela nivalis* and 15 *Sorex* spp. were trapped. The diversity index for small mammals trapped with Sherman traps and calculated for each trapping event separately ranged from 0 to 1.1 for the 23 trapping sites. In general, the number of small mammals trapped was low in 2008 and 2009 and, in comparison, 3.4- and 4.2-fold higher in 2010 (general annual trapping index 4.11 and 3.36, respectively, in comparison to 14.06, Table 1). The individual trapping index, calculated only for the PUUV-reservoir species *M.glareolus* for each trapping event, was from 0 up to 56.25 per site [62]. From the 353 trapped *M. glareolus* 349 were available for the investigation of PUUV antibodies as four animals escaped during trapping. Molecular analysis of RNA from all lung samples revealed S- and M-segment sequences with the strongest similarity to the local PUUV strain “Bavaria” [62]. The seroprevalence of PUUV was low in 2008 and 2009 and increased in 2010 (Table 1). Thus, annual PUUV seroprevalences increased in accordance with the annual trapping indices.

This indicates that the PUUV seroprevalence found during the epidemic year 2010 was much higher than both of the previous years. However, there might be a bias for the seroprevalence in 2008/2009 as the number of trapped animals and investigated sites was lower than in 2010. The high PUUV seroprevalence in bank voles found in 2010 is comparable to PUUV seroprevalences in bank voles found in several other studies in endemic years and areas in Germany. In previous studies, e.g., in the endemic years 2004 and 2005 at sites close to the BFNP PUUV prevalences from 21 to 34.5% and 45% were revealed [14,15,18]. From the 86PUUV-reactive *M.glareolus* six (7%) were juvenile animals that might have harbored maternal antibodies. Table 1 and Table 2 summarize the results on the trapping sites, animals and PUUV seroprevalence.

**Table 1:** Abundance of animals, trapping index, diversity index and PUUV seroprevalence in bank voles per year.

Parameter	2008	2009	2010
Number of trapped animals (n=674)	43	143	488
Number of trapping nights	9	40	30
General annual trapping index (calculated for all sites)	4.11	3.36	14.06
General annual diversity index of small mammals in Sherman traps (calculated for all sites)	1.01	1.37	0.83
Number of trapped Apodemus sp. (*A. flavicollis* and *A. sylvaticus*)	23	46	193
Number of trapped *Microtus agrestis*	4	19	12
Number of trapped Sorex sp.	1	10	4
Number of trapped *Glis glis*	0	8	0
Number of trapped *Mustela nivalis*	0	1	0
Number of trapped bank voles investigated for PUUV(total trapped number)	15 (19)	57 (57)	277 (277)
Annual PUUV seroprevalence [%] (number of PUUV-reactive bank voles)	6.7 (1)	7.0 (4)	29.2(81)

The seroprevalence of PUUV in bank voles was high in the BFNP and in some sites of the Donau (DO)-transect. PUUV seroprevalences varied between sites and between years of investigation ranging from 0% e.g., at 1318 m (T2_73) in 2010 to 60% at 517 m asl (Els5) in 2010, respectively.

It is known that bank voles are distributed unimodal over the altitude gradient [63]. We found that PUUV occurred at altitudes up to 1420 m, and to our knowledge this is the first time that PUUV has been recorded at high altitude. In comparison, the Swabian Alp is another important PUUV hot spot in Germany where altitudes reach 500 up to 1000 m asl [64,65].

### 2.2. Multivariate Analysis

Five of the eight potential predictors for the prevalence of PUUV-specific antibodies in bank voles were used for the final prediction model: (i) small mammal species diversity,(ii) mean annual temperature,(iii) mean annual solar radiation, (iv) herb layer coverage, and (v) deadwood layer coverage. The PUUV seroprevalence in bank voles increased with individual small mammal species diversity and deadwood layer coverage. Mean annual temperature, mean annual solar radiation, and herb layer coverage had a decreasing effect on the PUUV seroprevalence. The remaining three predictors (individual bank vole trapping index, shrub layer coverage, and beech coverage) were not associated with PUUV based on our analysis (*p*>0.1) and hence these variables were excluded from the final prediction model. For summary see Table 3.

**Table 2:** Environmental and climatic variables at the 23 trapping sites in the BFNP. As trapping indices and diversity indices change for each trapping event these are not shown in the table. * marks trapping sites of Donau (DO, Danube) transsect, ° marks sites where no PUUV seroreactivity could be found in *M. glareolus*.

Name of site	Altitude [m asl]	Mean annual temperature [°C] (1980-2007)	Mean annual solar radiation [kwh/m^2^]	Percent coverage of herb layer (0.02 ha)	Percent coverage of shrub layer (1 ha)	Percent coverage of deadwood layer (0.02 ha)	Percent coverage of beech upper layer (1 ha)
Isar32*°	317	8.26	3.16	80.0	30	2.0	0
Igg 35*°	379	8.03	2.68	50.0	30	5.0	30
Igg 33*°	412	7.45	3.99	3.0	2	7.0	45
Sal 27*	490	7.03	4.43	30.0	30	10.0	40
Els 5*	510	6.61	2.35	3.0	15	10.0	88
Els 8*	578	6.28	2.63	20.0	15	20.0	50
NP 37*	629	6.61	3.52	40.0	20	10.0	0
T4_29	670	6.34	3.37	30.0	0	10.0	0
T4_35	707	6.17	3.94	90.0	20	20.0	0
T4_39	767	5.98	3.71	0.5	30	5.0	15
T2_23	827	4.98	3.49	10.0	3	0.0	5
T4_47	894	5.43	4.09	20.0	30	3.0	0
T2_38	949	4.87	3.82	3.0	30	10.0	45
T2_44	990	5.20	3.70	20.0	10	20.0	50
T4_51	1007	5.02	3.55	80.0	10	20.0	25
T2_50	1082	5.31	4.25	20.0	10	20.0	20
T4_59	1150	4.62	3.08	80.0	10	10.0	0
T2_54	1184	5.07	4.33	30.0	0	40.0	0
T4_72°	1220	4.21	2.99	90.0	0	0.5	0
T4_78	1298	3.96	2.84	97.5	40	0.0	0
T2_73°	1318	4.32	3.52	97.5	0	10.0	0
T2_Wsh	1360	3.63	3.78	97.5	0	20.0	0
T2_67	1420	3.63	3.16	97.5	0	5.0	0

**Table 3:** Results of a GLMEM with a binomial distribution based on data from 23 sampling plots over three consecutive years (2008–2010). Abbreviation: CI: confidence interval.

Parameter	Log Odds Ratio†	95% CI	Z-value	*p*-value
Species diversity	1.156	(0.163, 2.148)	2.282	0.022
Deadwood layer coverage	0.040	(0.002, 0.077)	2.075	0.038
Mean annual solar radiation	-−0.758	(−1.449, -(−0.066)	−2.148	0.032
Mean annual temperature	-−0.501	(-(−0.873, -(−0.129)	-−2.641	0.008
Herb layer coverage	-−0.018	(-(−0.029, -(−008)	-−3.368	0.001
Shrub layer coverage	0.011	(-(−0.022, 0.043)	0.651	0.515
Beech coverage	-−0.002	(-(−0.024, 0.021)	-−0.153	0.878
Trapping index	0.005	(-(−0.024, 0.035)	0.338	0.735

† Each variable included in the model as a linear term (*i.e.*, the log odds ratio corresponds to a 1 unit increase in the variable). The variables are defined in Table 4.

In our analyses, in the BFNP the main positive predictor for PUUV seroprevalence was small mammal species diversity. There are two distinct theories to explain the relationship between species diversity and pathogen prevalence: the “Dilution Effect” and the “Amplification” or “Rescue Effect”. The “Dilution Effect” postulates that high species diversity decreases pathogen prevalence by decreasing host density, reducing encounters between hosts, altering host behaviour, or reducing host survival [41,66,67,68,69,70,71,72,73]. On the contrary, the theory of “Amplification” suggests that increased pathogen prevalence is associated with high species diversity [74,75]. For emerging infectious diseases it has been suggested that balanced ecosystems with a higher biodiversity have a reduced pathogen prevalence [75]. Our results suggest that an “Amplification Effect” is present in the investigated areas in the BFNP. So, community composition might be most critical for the virus prevalence. However, *M. glareolus* is the only host for PUUV and spill-over to other species is a rare event [14,76]. We found onlyone spill-over to *Apodemus flavicollis* at one trapping point of the Danube transsect (Elsenthal 5, Els5) in 2010,where 60% (n=6/10) of the investigated *M. glareolus* were seroreactive*.* In this *A flavicollis* seroreactivity was shown but this animal did not carry PUUV RNA [62].A positive effect of biodiversity on PUUV seroprevalence is in contrast to findings for hantaviruses other than PUUV such as Sin Nombre-, Choclo-, Laguna Negra, and Bayou virus where a negative relationship between rodent or small mammal species diversity and hantavirus prevalence has been documented [66,72,74,77,78]. However, for American hantaviruses the picture might be more complex as there are potentially several reservoir species and transmission might occur between species [68,77]. Here small mammal communities are less diverse in Central Europe than in North America. Thus for Central Europe, further studies are needed to confirm the “Amplification Effect” in other PUUV hotspots. These studies could also investigate possible mechanisms for the association between PUUV and biodiversity, e.g., different contact rates between bank voles and other small mammal species might affect habitat usage, social behaviour, and aggression of bank voles.

The second factor that was positively associated with PUUV seroprevalence in bank voles was deadwood. To the authors’ knowledge, the influence of deadwood on hantavirus prevalence has not been investigated elsewhere. In general, the appearance of small animals such as bank voles seems to be positively correlated with deadwood environments [44,79]. Deadwood might increase available food resources e.g., fungi growing on it, provide shelter during roaming, and may be associated with higher rodent population densities, which might trigger hantavirus epidemics [40,80].

Annual solar radiation was negatively associated with PUUV seroprevalence. This is the first study that has examined this association and it might be explained by the effect of UV radiation on the stability of the virus. For example Hantaan virus was shown to be inactivated by UV irradiation for 3 minutes at 312 nm (1.4 J/cm^2^) [32]. It has also been suggested that fluctuations in the densities of common vole populations, and hence hantavirus prevalence, might be correlated with the duration of sunshine [81]. The impact of sunshine and UV light on the stability of PUUV in a natural surrounding has to be investigated in more detail as UV light might not reach areas where the virus is present e.g., in/on voles, on the ground, vegetation, or in nests.

Temperature seems to be another important—but negative—factor in our investigation. It has been suggested that low temperatures might positively influence viral stability [82,83]. Temperature might not only affect the stability of the virus, but also the host itself. Bank vole growth may be sensitive to mild winter conditions if they have a negative impact on habitat of the rodents [84]. In another study, snowfall in December and in April was associated with high host population densities [81]. Alternatively, mild winters and springs could positively influence rodent survival rates and food supply and thus affect rodent population dynamics [34,35,85].

Other studies have found that certain properties of the voles’ habitat, e.g., forest patches and dense ground vegetation, are associated with the likelihood of hantavirus disease by either influencing the abundance of voles or by allowing the virus to persist in the environment [19,24,40,48,80,86]. Interestingly, in our investigation herb layer coverage was the factor with the highest negative impact whereas shrub layer coverage was not significant (*p*=0.515). Both herb and shrub layer give shelter and several plants may be a source for nutrition, but will also have an influence on moisture, light and biodiversity [87,88]. In general the herb layer contains more non-wooden plants such as grass or herb species, these favour rodents such as *Microtus agrestis* in forests, while *M. glareolus* occurs is usually found in beech forests with heavy tree seeds [54,88,89]. These are more dominated by less cover near ground. Thus, a general lower attractiveness of grass dominated forest patches for *M. glareolus* may explain a general lower density and therefore lower prevalence [53,88,89].

So far—to the authors best knowledge—in PUUV studies shrub und herb were not differentiated since details on habitats are rarely shown [46,90,91]. It has been reported that NE occurs infrequently in the PUUV hotspots of South-western Germany where the primary land cover is coniferous forest [35]. In comparison, North American hantaviruses (e.g., Sin Nombre virus) are transmitted by sigmotondine rodent species, which are found in shrubland and grassland. And, humans living close to these habitats were at highest risk for acquiring hantavirus pulmonary syndrome (HPS) [92,93]. Thus, further studies are needed to compare the influence of different plant layers on the prevalence of PUUV in bank voles and the frequency of human disease cases. 

In our study the seroprevalence of PUUV was not associated with bank vole population density (*p* = 0.735). Some of the investigations of PUUV in bank voles e.g., in 2010 were performed in a hantavirus outbreak year. Herein, higher trapping indices despite the differences in trapping times and numbers per year were shown. It has been suggested that transmission of PUUV increases with the density of the carrier population and that this is therefore an important predictor of hantavirus outbreaks in Europe [26,48,80,94]. But other authors have proposed that factors other than population density e.g., environmental, climatic and social conditions drive high PUUV prevalences in epizootics [19,25,40,41]. Our study confirms that environmental and climatic factors determine PUUV transmission. The behaviour and ecology of PUUV reservoirs will have to be further studied, in order to predict future outbreaks and minimize the risk of human infection [70,95,96].

Finally, in contrast to other studies in Sweden, Finland, Belgium, Baden-Wuerttemberg/South-Western Germany [21,34,35,45,46,47,48] beech cover layer was not a significant predictor of PUUV seroprevalence in bank voles our study (*p*=0.878). Interestingly, there was beech mast in 2009 in the BFNP (*ca.* 680kk/ha dried beech fruit). However, the distribution of beech trees over the investigated area is variable. Broadleaf trees are found up to 1150 m in the BFNP and woodrush beech forest is the dominant habitat in the BFNP [97]. In Lower Saxony, North-Western Germany, a study over 16 years showed that beech had a positive effect on the vole population [98]. Investigations performed between 2001 and 2007 in Baden-Wuerttemberg, Germany, also showed that beechnut mast beneath beech forest influenced the annual and regional variation in hantavirus outbreaks [34,35]. Despite several studies showing the impact of beech mast on bank vole populations, the association of beech abundance and beech mast and PUUV risk is controversial (for review see [12]). Bank voles have a varied nutrition including seeds, herbs, berries, fungi, and also insects [89]. Therefore, the availability of beech cannot be the only factor explaining the prevalence of PUUV and human disease cases.

### 2.3. IllustrativeMap of Puumala Virus Prediction in Bank Voles

**Figure 2:**
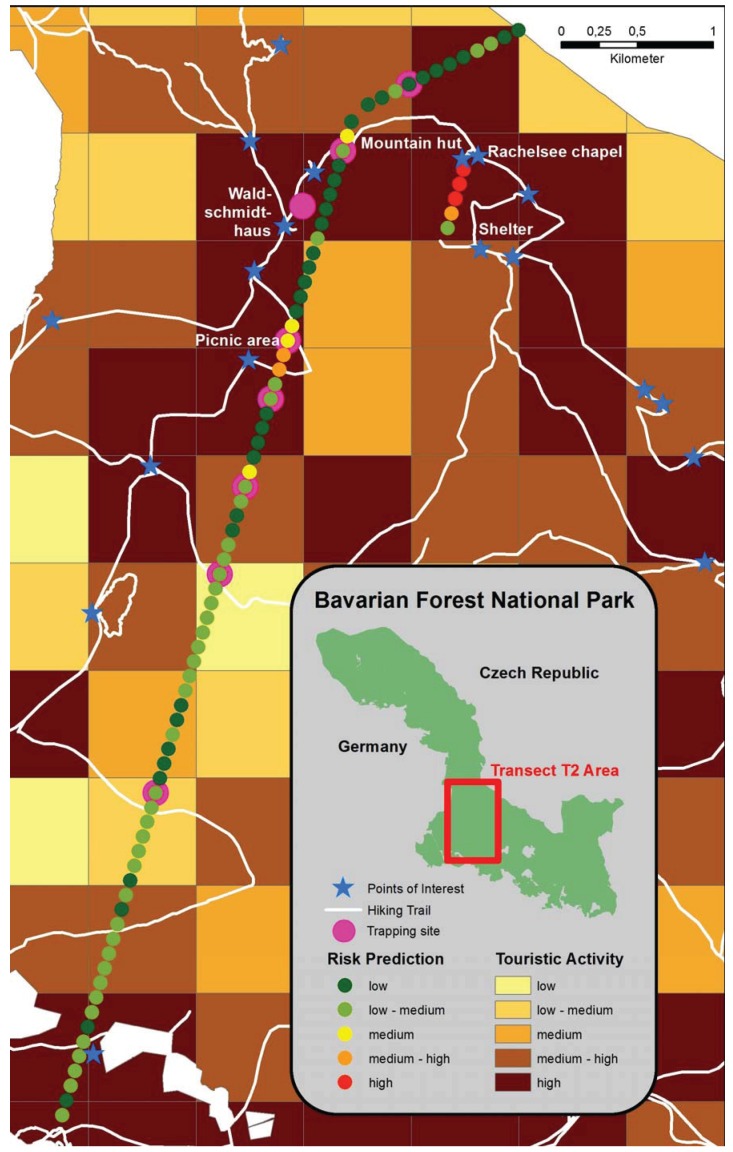
Illustrative risk prediction map for PUUV in bank voles in transect T2.

The prevalence of PUUV in rodents was predicted in 330 plots of the four transects in the BFNP using data on species diversity, dead wood coverage, herb coverage, mean annual temperature, mean solar radiation. An ArcGis database was used to produce an illustrative risk map for the probability of PUUV prevalence in *M. glareolus* along the altitude gradient T2 (Figure 2 and Supplementary Figure S2). The probability of PUUV prevalence in bank voles was classified as ‘low’ (0.5%-15.5%), ‘low-medium’ (15.6%–30.6%), ‘medium’(30.7%–45.6%), ‘medium-high’(45.7%–60.7%), and ‘high’(60.8%–75.7%). The map also shows levels of touristic activity [99](‘low’, ‘low-medium’, ‘medium’, ‘medium-high’, and ‘high’), and points of interest, e.g., huts are highlighted where humans and PUUV carriers might come into closer contact. The map shows that areas with a high tourist activity, such as the alpine hut Waldschmidthaus, the Rachel chapel and the picnic area are also areas with ‘medium-high’ and ‘high’ hantavirus prevalence in the rodent (Figure 2).

The study area has a low human population density, but in some regions there is a high touristic activity. In general, there is increasing contact between people and wildlife in Germany. For example, there has been a trend among the working population to shift recreational activities to natural settings and this has made people more vulnerable to zoonotic pathogens [100]. There is a clear link between human behaviour and human population density and risk of PUUV infection [35,58,101,102,103]. The risk of human infection is correlated with the prevalence of PUUV in bank voles [104]. Several studies have shown that exposure in forests and outdoor leisure activities such as gardening, collecting mushrooms, visiting a forest shelter are risk factors for human infection [102,105]. Further, professions that involve prolonged stays in forested areas (e.g., lumberjacks and woodsman)are at a higher risk for PUUV infection [106,107]. Sero-epidemiological studies on people working in the BFNP are needed to validate the risk map.

Our study had two objectives:(1) to identify predictors of PUUV seroprevalence in bank voles based on the characteristics of bank vole populations and their environment (e.g., climate and forest structure), and (2) to generate a risk map of PUUV prevalence. The map that we have produced is based on data collected over three years. Ideally, future maps should use at least 10-years of trapping-data to allow for cyclical variation in PUUV prevalence. 

## 3. Experimental Section

### 3.1. The Study Area

The BFNP (242 square kilometers) is situated in the centre of the German part of the Bohemian Massif (the so-called Inner Bavarian Forest), and, together with the adjacent Czech Sumava National Park, forms one of the most extensive, contiguous, and homogeneous forest landscapes in Central Europe. Ninety-eight percent of the BFNP is covered by forest [108]. The highest elevations of this low mountain range area are found along the Czech-German border and form the watershed between the Danube and Elbe catchment areas. The National Park is characterized by montane and subalpine areas with a vertical range of approximately 800 m. Slopes in the National Park have mainly a southwest exposition. Long-term phases of weathering and erosion have led to rounded landforms. On a larger scale, the Bavarian Forest lies in the temperate zone and the climate is characterized by Atlantic and continental influences. Total precipitation is between 1200 and 1800 mm per year, depending on the altitude. The annual mean air temperature varies from 5.1 °C in valley sites influenced by inversion effects, 5.8 °C on hillsides, and 3.8 °C in the higher montane zones [109,110]. Above about 1100–1200 m asl, the high montane forest is dominated by Norway Spruce (*Picea abies*), with a low proportion of European beech (*Fagus sylvatica*) and mountain ash (*Sorbus aucuparia*); below this altitude, the mixed montane forest is dominated by spruce, beech, and silver fir (*Abies alba*) [111].

### 3.2. Trapping Sites and Collection of Small Animals

Trapping sites derive from three independent transects (T2, T4, DO, see Table 2, Supplementary Figure S1) which were selected out of approximately 400 transection sites in the BFNP originating from the BIOKLIM project [60]. Transects T2 and T4 were selected as being typical of the BFNP. Transect T2 extends from 827 m to 1420 m asl along the mountain Rachel. An additional site, the Waldschmidthaus (T2_Wsh), which is an alpine hut located at 1360 m asl and frequently visited by hikers, was included. Transect T4 extends from 629 m to 1298 m asl along the mountain Lackenberg. Transect DO (“Donau”, Danube river) extends from 317 m to 578 m asl. Trapping was performed from spring to autumn, because in winter high snow cover makes the soil inaccessible. Furthermore, because of animal welfare trapping was not allowed in months without snow. In summary, 23 sites were selected for animal collection in the BFNP -15 sites in September and October 2008, 20 sites in May to October 2009, and 23 sites in May to October 2010—along an altitude gradient ranging from 317up to 1420 m asl. The plot sizes were 18 ×18 m and were mapped using GPS receiver (Garmin, Olathe, KS,USA). Each trapping grid was laid out with 4 ×4 (n=16) traps in 6meterintervals. Small mammals were captured using Sherman Traps™ (H. B. Sherman-Traps Inc., Tallahassee, FL, USA) which were equipped with either small pieces of Golden Delicious apple or Nutella® as bait and laid out in the evenings [14,61]. Traps were checked twice on two successive days. Captured small mammals were anaesthetised using isoflurane (Actavis Dtl. GmbH & Co. KG, Munich, Germany) and blood was collected in serum vacutainers (Becton, Dickinson and Company, Franklin Lakes, NJ, USA) by heart puncture using cannulas (BD Microlance, Becton, Dickinson and Company). Subsequently, all animals were euthanized in accordance with the German Animal Protection Act. The trapping site was recorded and small mammals were kept at −40 °C until section. During necropsy transsudates were collected which served as substitute when serum could not be obtained.

### 3.3. Serological Investigation of Bank Voles for PUUV

Antibodies against PUUV were determined in bank vole sera (1:10 diluted in PBS) and/or transsudates (undiluted) using an indirect immunofluorescence test (IIFT) assay (Progen Biotechnik, Heidelberg, Germany) consisting of cell cultures infected with PUUV spotted on multi-well glass slides according to the manufacturer’s instructions [14]. In brief, sera were incubated for 30 min on the slides. After washing three times with PBS-Tween (1%), fluorescein isothiocyanate(FITC)-conjugated goat anti-mouse immunoglobulin G(IgG) (BIOZOL, Eching, Germany) was used as secondary antibody and incubated for 30 minutes. Washing three times in PBS was followed by counterstaining with Evans Blue (bioMérieux, Nürtingen, Germany). Slides were examined using a fluorescence microscope (Nikon, Düsseldorf, Germany) by two independent examiners with equal results. Detection of spotted green fluorescence in the cytoplasm was considered a reactive sample. Positive and negative controls (previously investigated bank vole sera confirmed in PUUV ELISA [14]) were used for each slide. 

### 3.4. Choice of Predictors of PUUV Seroprevalence

Data on environmental and climatic variables were derived from the BIOKLIM project where extensive data on macroclimate and vegetation was collected and analysed for each of the 400 transection sites in the entire BFNP [60].In addition, some data (e.g., small mammals’ diversity index) were used from the longitudinal study over three consecutive years. In summary, eight continuous variables, either host-associated (trapping index, small mammal diversity index), climatic (mean annual solar radiation, mean annual temperature) or habitat-specific (herb layer coverage, shrub layer coverage, beech layer coverage, deadwood layer coverage), were selected to identify associations with PUUV prevalence (Table 4).

**Table 4:** Description of the variables within the data set (climate and vegetation composition) used to predict PUUV prevalence in bank voles along two altitude gradients in the BFNP in a GLMEM.

Variable	Explanation
Trapping index	Number of bank voles/100 trapping nights (2008–2010, individually calculated for each site for each trapping event)
Species diversity	Shannon‘s diversity index of small mammals trapped with Sherman live traps (2008–2010, specifically calculated for each site for each trapping event)
Mean annual temperature	Mean annual temperature in °C (1980–2007)
Mean annual solar radiation	Mean annual solar radiation in kwh per m^2^
Herb layer coverage	Percent coverage of herb layer (0.02 ha) recorded on 1 m altitude
Shrub layer coverage	Percent coverage of shrub layer (0.02 ha) recorded on 1–5 m altitude
Beech layer coverage	Percent coverage of beech upper layer (1 ha) recorded on >15 m altitude
Deadwood layer coverage	Percent coverage of deadwood layer (0.1ha)

#### 3.4.1. Trapping index

The trapping index was used as a parameter to indicate the relative small mammal population density for each trapping site or year. Herein it was calculated as the total number of *M. glareolus* caught in Sherman traps divided by the number of trap-nights and expressed as the number caught per 100 trap-nights. Data were collected on two consecutive nights at each site [27].General annual trapping indices were calculated for general comparisons (Table 1). For the statistical analysis and modelling individual trapping indices were calculated for each trapping event for each site *i.e.*, for two subsequent trapping nights.

#### 3.4.2. Small mammals’ diversity index

The diversity of species of all small mammals trapped with Sherman traps was investigated using the Shannon diversity index [112,113]. It is calculated from the number of individuals (N)in different species, and the number, n_i_,of individuals in species i. The Shannon diversity index was calculated for each site as H = −Σp_i_ × ln (p_i_), where p_i_ = n_i_/N. The Shannon diversity index for a given number of species reaches a maximum if all species occur in equal abundances. For the statistical analysis and modelling individual Shannon indices were calculated for each trapping event for each site i.e. for two subsequent trapping nights.

#### 3.4.3. Mean Annual Solar Radiation

The mean potential solar radiation (kWh/m^2^) was calculated from a digital terrain model SAGA Geographical Information Systems (GIS) with the module “Incoming Solar Radiation” and values represent the potential radiation as derived from the topography [114].

#### 3.4.4. Mean Annual Temperature

In this study, time series available for the study region based on data from 14 main climate stations, 88 rain gauges, and 13 temperature and humidity loggers were used. Additionally, 30 data loggers were set up on representative sites along the transects and a statistical model predicting the daily means of temperature for each plot for the time span between 1980 and 2007, using the digital terrain model ArcEGMO, was applied [115].

#### 3.4.5. Herblayer

Data on herb layer (% cover horizontal projection) in the BFNP was taken from one relevee with a size of 0.02 ha in the centre of the small mammal plot. All plants up to 1m were recorded [60].

#### 3.4.6. Shrub Layer

Data on shrub layer (% cover horizontal projection) in the BFNP was taken from one relevee with a size of 0.02 ha in the centre of the small mammal plot. All plants from 1 to 5 m were recorded [60].

#### 3.4.7. Beech Layer Coverage

Data on beech trees >15 m in the BFNP was derived from records of the area covered by mature beech trees per ha (%) in 2006 [60].

#### 3.4.8. Dead wood layer coverage

Data from 2006-estimates of areas in the BFNP covered by lying dead wood on 0.1 ha were used [60].

### 3.5. Data Analyses

A Generalised Linear Mixed Effects Model (GLMEM) based on a binomial distribution with a logit link function was used to predict the seroprevalence of PUUV in bank voles. The model was fitted to data collected in three consecutive years (2008, 2009, 2010) using the function “glmer” in the “lme4”packagein R [116,117]). The initial model included eight predictors as fixed effects: (i) small mammal species diversity,(ii) mean annual temperature,(iii) mean annual solar radiation, (iv) herb layer coverage, (v) deadwood layer coverage,(vi) shrub layer coverage, (vii) beech coverage, and (viii) trapping index. To account for repeated measures at the same site we included trapping site as a random effect. All predictors with a *p*-value <0.1 were included in the final prediction model. The predicted prevalence of PUUV in bank voles was calculated at along one transect (e.g., 85 points at T2), using the fixed effects parameter estimates obtained from the model(function “plogis” in the “lme4” package, R Core Development Team 2013 [116,117]).An illustrative map indicating the probability of PUUV in bank voles along one transect together with data indicating touristic activity and points of interest(including summer activity for tourists) was generated using ArcGIS™ [118].

## 4. Conclusions

In this study we investigated the effects of population and environmental factors on the seroprevalence of PUUV in bank voles in the BFNP, a region with increasing human hantavirus cases and with intense outdoor recreation. PUUV seroprevalences varied in the investigation years and was positively associated with the species diversity and deadwood layer, and negatively associated with mean annual temperature, mean annual solar radiation, and herb layer. An illustrative risk map was generated to predict PUUV seroprevalence in bank voles in a transect of the national park. 

Our data may give further insight into the microevolution of PUUV, to identify factors influencing the dynamics of infection foci and transmission paths, as hantaviruses are not homogenously distributed in the host population [119]. Although the host-ecology conditions may differ from other hantaviruses and the list of factors driving hantavirus transmission may still be far from complete, our findings provide valuable information and encourage further studies in the field. Further the information provided in the PUUV risk map might be useful in the management of the BFNP. This may help to promote the prevention of PUUV infections as no vaccine and no specific treatment is currently available [120].

## Supplementary Files

Supplementary File 1
