# Supplementary material for: Identification of FactorsInfluencing the Puumala Virus Seroprevalence within Its Reservoir in aMontane Forest Environment
Source: Viruses. 2014 Oct 23;6(10):3944–67. doi: 10.3390/v6103944 (PMC4213572; doi:10.3390/v6103944)
Supplement: Supplementary File 1 [file viruses-06-03944-s001.pdf]

Supplementary Materials

Identification of Factors Influencing the Puumala Virus Seroprevalence within Its Reservoir in a Montane Forest Environment

Bryan R. Thoma, Jörg Müller, Claus Bässler, Enrico Georgi, Anja Osterberg, Susanne Schex, Christian Bottomley and Sandra S. Essbauer

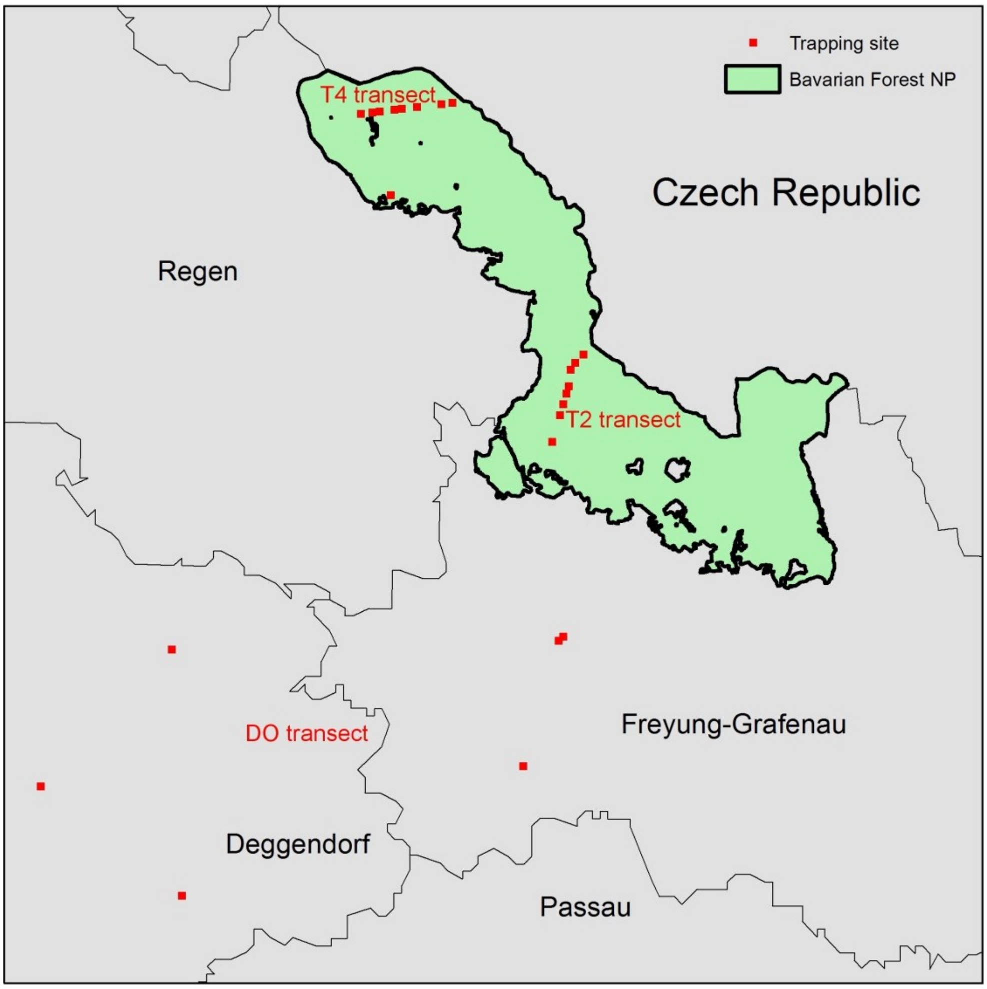

Supplementary Figure S1. Trapping sites along three transsects in the BFNP.

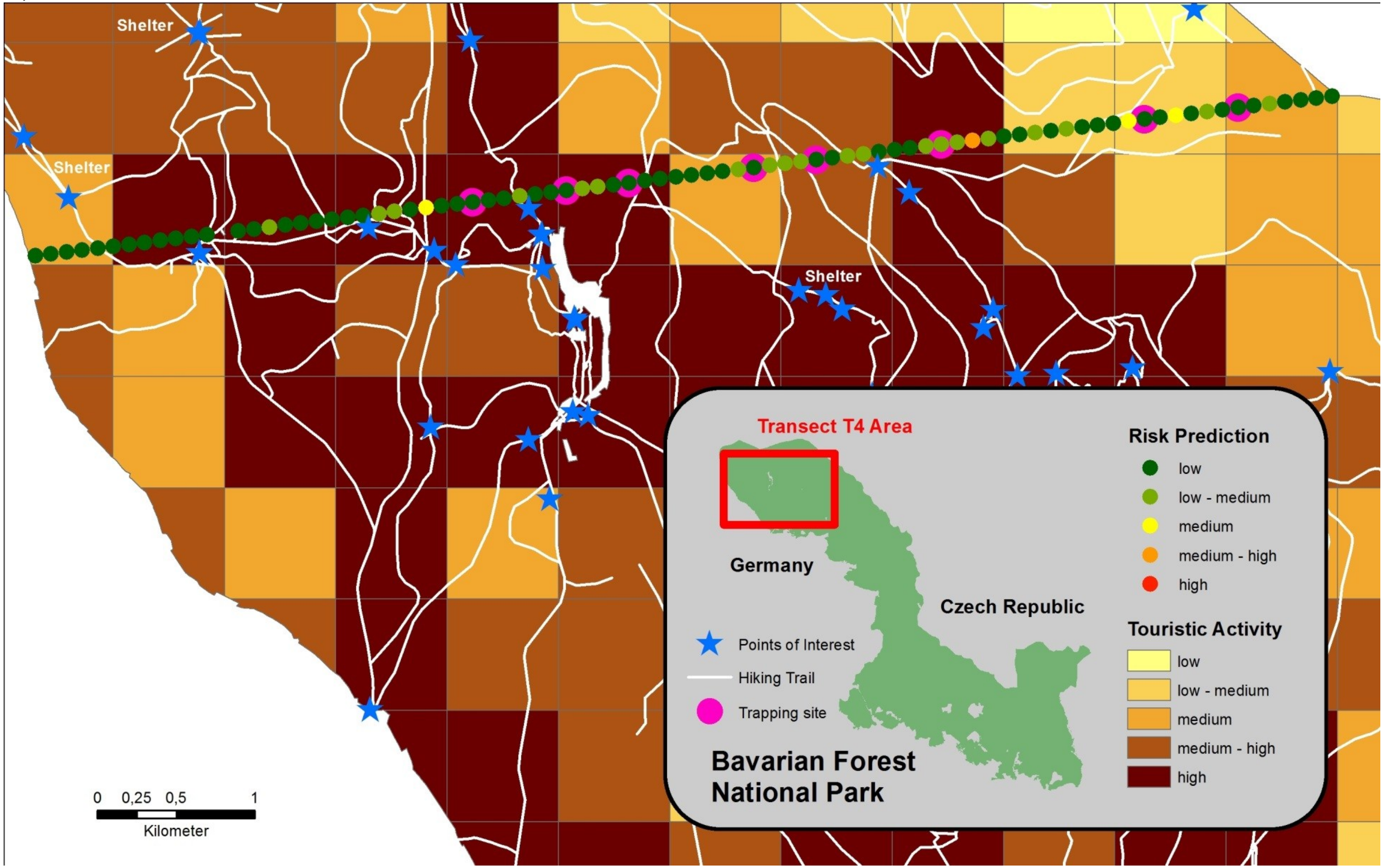

Supplementary Figure S2. Illustrative risk prediction map for PUUV in bank voles at transect T4.
